# Spatial variations and pools of non-structural carbohydrates in young *Catalpa bungei* undergoing different fertilization regimes

**DOI:** 10.3389/fpls.2022.1010178

**Published:** 2022-09-29

**Authors:** Zhuizhui Guan, Qifeng Lu, Yubiao Lin, Daiyi Lin, Yizeng Lu, Qingjun Han, Ningning Li, Wenjun Ma, Junhui Wang, Yan Su, Jiyue Li, Quan Qiu, Qian He

**Affiliations:** ^1^ Guangdong Key Laboratory for Innovative Development and Utilization of Forest Plant Germplasm, College of Forestry and Landscape Architecture, South China Agricultural University, Guangzhou, China; ^2^ Shandong Provincial Center of Forest and Grass Germplasm Resources, Jinan, China; ^3^ Institute of Resource Cultivation, Research Institute of Forestry, Chinese Academy of Forestry, Beijing, China

**Keywords:** spatial variation, non-structure carbohydrate (NSC), sugar and starch pool, carbon allocation, integration of water and fertilizer

## Abstract

Despite the importance of non-structural carbohydrates (NSC) for growth and survival in woody plants, we know little about whole-tree NSC storage. Here, *Catalpa bungei* trees fertilized using different schedules, including water and fertilizer integration, hole application, and no fertilization, were used to measure the spatial variations of sugar, starch, and NSC concentrations in the leaf, branch, stem, bark, and root. By calculating the volume of whole-tree NSC pools and the contribution of distinct organs, we were also able to compare the storage under various fertilization regimes. We found that the spatial distribution patterns of each organ undergoing different fertilization regimes were remarkably similar. Height-related increases in the sugar and NSC concentrations of the leaf and bark were observed. The concentrations of sugar and NSC in the branch did not appear to vary longitudinally or horizontally. The sugar and NSC concentrations in the stem fluctuated with height, first falling and then rising. The coarse root contained larger amounts of NSC components in comparison to fine root. Contrary to no fertilization, fertilization enhanced the distribution ratio of the leaf, branch, and stem NSC pools while decreasing the distribution ratio of the root NSC pool. Particularly, the addition of fertilizer and water significantly increased the biomass of the organs, enhancing the carbon sink of each organ and whole-tree in comparison to other fertilization regimes. Our main goal was to strengthen the empirical groundwork for comprehending the functional significance of NSC allocation and stock variations at the organ-level of *C. bungei* trees.

## Introduction

In trees, non-structural carbohydrates (NSC) constitute a crucial but poorly measured carbon reserve. Starch and soluble sugars, which are interconvertible and work with current photoassimilates to supply energy and carbon substrate for metabolic processes, make up the majority of non-structural carbohydrates (Chapin et al., 1990). The concentrations of NSC can indicate both the plants’ growth condition and the allocation strategy used during seasonal dynamics (Körner, 2003). The tree germination in spring (Schädel et al., 2009), respiration in winter (Ögren, 1999), alleviation of water stress (Sala et al., 2012; Dong and Li, 2013), and shoot sprouting after cutting are closely related to the level of plant NSC stocks in temperate forest ecosystems (Bond and Midgley, 2001).

It is widely acknowledged that the NSC concentrations change with the seasons, organs, and species (Gholz and Cropper, 1991; Richardson et al., 2013; Richardson et al., 2015). Due to the spatial heterogeneity of NSC concentrations in the trees with varying growth phenology, it is challenging to estimate the NSC stocks of the stand from the perspective of each individual tree (Barbaroux et al., 2003; Woodruff and Meinzer, 2011). According to the majority of research, canopy location affects the NSC concentrations of leaf and branch (Barbaroux et al., 2003; Landhauser and Lieffers, 2003; Li et al., 2009; Yan et al., 2012). However, there are rare cases of changes to the horizontal densities of branch NSC. (Barbaroux et al., 2003). Several researchers have systematically monitored radial and longitudinal fluctuations of truck NSC concentrations (Barbaroux et al., 2003; Hoch et al., 2003; Landhauser and Lieffers, 2003; Würth et al., 2005). However, the methods to accurately calculate organ carbon pool are not completely apparent. For example, allometric equations are used to calculate carbon reserves of tree organ NSC concentrations and biomass (Barbaroux et al., 2003; Zhang et al., 2014; Furze et al., 2019). The anticipated value of the organ carbon pool, however, might differ from the actual value because the biomass estimated by the models could have some degree of precision deviation. Thus, the organ biomass obtained from tree dissection is needed to precisely assess tree carbon pools.

An important silviculture technique for managing forests is fertilization. Plant photosynthesis can be altered by fertilization, which will then have an impact on NSC concentrations and distribution patterns (Chen et al., 2021). According to the ecological stoichiometry concept, an organism’s construction incorporates the carbon, nitrogen, phosphorus, and other components necessary for plant growth in a particular ratio (Ma et al., 2017). Consequently, the presence of additional nitrogen, phosphorus, and other nutrients in plants may cause them to use more carbon for biological construction, which would lead to the consumption of NSC pools (Xie et al., 2018). Although the effects of nutrient input on plant NSC dynamics have been a fascinating subject for several decades (Zheng et al., 2010; Guo et al., 2016; Wang et al., 2016; Zhao and Gong, 2020), it is still unclear how different fertilization regimes affect the size and distribution patterns of tree NSC pools. The advantages of combining water and fertilizer include increased water and nutrient efficiency, increased crop output, and improved crop product quality. In contrast to agricultural environments (Liu et al., 2020; Zou et al., 2020; Sun et al., 2022), forestry ecosystems hardly ever use this water-fertilizers strategy. It is yet unknown whether this approach has the same impact on tree carbon storage when compared to other fertilization regimes.

A precious and superior-quality timber tree, *Catalpa bungei* is traditionally grown in China’s warm-temperate and subtropical regions. Here, we characterize whole-tree NSC storage in three fertilization regimes and investigate the spatial variations in organ NSC concentrations. We specifically addressed the ensuing issues: (1) Do various fertilization regimens have an impact on the spatial variations of organ NSC concentrations? And if so, what is the impact? (2) What are the whole-tree NSC pools for each of the three fertilization regimens? (3) How much do particular organs contribute to whole-tree storage in different fertilization regimes? Our research will strengthen the empirical foundation for comprehending the functional significance of NSC stock variations and allocation at the whole-tree and organ levels.

## Materials and methods

### Study site and species

The experiment was carried out at the Jujube Preservation Warehouse in Zhangqiu City, Shandong Province, China (36°25′-37°09′ N, 117°10′-117°35′ E). A moderate monsoon climate prevails at the location, with a mean annual temperature of 12.8°C, a maximum mean monthly temperature of 27.2°C (July), and a minimum mean monthly temperature of -3.2°C (January). The average annual precipitation is 600.8 mm, the frost-free period is 192 days, and there are 2647.6 hours of sunlight each year. The *C. bungei* has a 130–150 day growing season that lasts from May to September.

Pure plantations of *C. bungei* were chosen, and they were started in a planting grid measuring 3.0 x 4.0 meters in March 2017 using 2-year-old clonal seedlings (“9-1”). There were 18 plots in all, with a planting area of 0.8 hectares, and 45 trees were planted in each plot (5 rows and 9 columns). Since planting, there had been no tending. After planting, the mean tree height and diameter at breast height (DBH) were 4.2 m and 4.0 cm, respectively. Immediately following afforestation, the soil’s physicochemical characteristics, including pH (7.67), soil organic matter (19.64 g/kg), total N (0.91 g/kg), total P (0.53 g/kg), total K (16.7 g/kg), alkaline hydrolyzed N (81.88 mg/kg), available P (32.10 mg/kg), and available K (176.82 mg/kg) were measured.

### Fertilization

We carried out a split-plot experiment in early May 2018 and randomly picked 9 plots for fertilization. Each plot had a size of approximately 384 square meters. We created three fertilization strategies: hole fertilization (HF), water and fertilizer integration (WF), and no fertilization (CK). Three plots were put up with isolation zones between each fertilization regime, and each plot received one fertilization regime at random. Six to eight meters separated the plots. In May 2018, the first fertilization was administered. The fertilizer used was N (24 g/tree), P_2_O_5_ (8 g/tree), and K_2_O (16 g/tree). The total amount of fertilizer applied each year was the same for WF and HF. Starting in the second year, the total amount of fertilizer sprayed annually was increased by 20% as compared to the previous year. All fertilizers were applied once as part of the HF treatment in May. On the south and north sides of the trunk, we excavated a hole that was 20 cm in diameter and 30 cm deep. We then split the fertilizer into two equal portions and poured it into the holes. Each year’s supply of WF fertilizer was divided into 12 equally sized portions, each of which was applied once every 10 days (d) beginning on May 1 and ending on September 1. The WF used sophisticated drip irrigation equipment to apply fertilizers to plant roots after dissolving fertilizers in 1000 L of water (HN-BXE, Huinong Automation Company, China). For HF and WF, urea was utilized as a N fertilizer and potassium sulfate as a K fertilizer. Superphosphate was the P fertilizer used for HF, whereas ammonium dihydrogen phosphate was utilized for WF. The Taobao website was the only place where fertilizer could be purchased.

The DBH of 15 randomly chosen trees from each plot was measured and recorded at the end of the growing season from 2018 to 2021. In October 2021, we measured the tree height, DBH, and crown width of trees receiving various fertilizer regimens (**Table S1**). **Figure S1** showed data on air temperature and precipitation collected by a local meteorological station for the years 2017 through 2021.

### Field sampling

In October 2021, we chose and cut down two healthy trees from each plot, resulting in 6 trees for each fertilization treatment. The sample trees displayed the following traits: a single, straight stem, an open-growing crown, and no indications of illness or epicormic growth. We measured the height, DBH, and crown diameter of the downed trees (**Table S2**). Each tree was separated by the following organs: leaves, branches, stem, stem bark, and coarse and fine roots. Each organ’s fresh weight and dry weight (biomass) was measured in the field to the nearest 10 g (dead branches were excluded as a very minor component). When the tree was chopped down, we split the height of the trunk into five equal parts (H1–H5; H1, lowest part; H5, uppermost part; see **Figure S2**) in order to quantify the longitudinal variation of the stem and bark NSC. A standard 4.3-mm increment bore (Haglöf Company Group, Lngsele, Sweden) was used to collect the stem core up to the pith at five heights along the south side of the trunk. The bark samples were removed from the location of the stem core with a knife. The biomass of bark was not acquired because there was not much bark left over after taking a sample, and it was discarded. We split the length of the crown into five equal sections (h1-h5; h1, lowest part; h5, topmost part; see **Figure S2**), and harvested a standard branch from each section in order to examine longitudinal variation in leaf and branch NSC. In the meantime, we gathered five healthy leaves off the normal branch’s tip. We divided the length of the sample branches into 4 equal portions (P1-P4; P1, proximal part; P4, distal part), and cut the branch discs about 3 cm thick in each part with branch shears in order to study the horizontal variation of the branch NSC. An excavator was used to dig out all of the roots, and the soil was gently scooped out with a spade. According to root diameters, the roots were separated into coarse roots (>2 mm) and fine roots (≤2 mm). To get rid of soil particles, all plant materials were vigorously brushed. To quantify the tissue moisture content of each organ, representative samples were also gathered. The measured NSC samples were transferred to the lab in less than two hours using dry ice, and then destroyed in a microwave oven for five minutes at an 800 W power level (Würth et al., 2005). The slain samples were dried for 72 hours at 65°C in a convection oven. To compute the percentage of dry weight of the tissue, the samples with the measured tissue moisture content were weighed right away and subsequently dried to a consistent weight at 65°C. Five leaf samples, twenty branch samples, five stem samples, five bark samples, and two root samples made up the total amount of samples used to determine each tree’s NSC.

### NSC analyses

All dried samples were ground to a 0.2 mm sieve size with a high-speed pulverizer and then stored in plastic bags before NSC was analyzed. Soluble sugar (measured as the sum of glucose, sucrose, and fructose) and starch concentrations were determined by using with an anthrone sulfuric acid method (Morris, 1948). Briefly, wood powder (200 mg) was extracted with 7 ml of 80% ethanol first in an 80 °C bath for 30 min. Three parallel extractions were made for each wood sample. After the extraction process, the samples were centrifuged in a high-speed centrifuge (3,000 rpm, 10 min; 5810R, Eppendorf, Germany). After reaction of supernatant with anthrone, soluble sugar extract was measured with a microplate reader at 620 nm (Ratastie 2, FI-01620 Vantaa, Finland). The ethanol-extracted residue was boiled in hot water for 15 min, solubilized in perchloric acid, followed by three centrifugations. After reaction of supernatant with anthrone, starch extract was measured with a microplate reader at 620 nm. Starch concentrations were determined using a starch standard curve, while soluble sugar concentrations were determined using a sucrose standard curve. The NSC concentration was calculated as the sum of soluble sugar and starch and expressed on a dry weight basis (% dry weight).

### Statistical analysis

First, the fresh weight of each organ (leaf, branch, stem, and root) was multiplied by the corresponding organ’s water content to get the tissue’s water content; Then, the difference between the fresh weight and the tissue’s water content was used to calculate each organ’s dry weight. Next, the mean of the sugar concentration in the leaf (or branch) of the five canopy levels was determined; Consequently, the mean of the leaf (or branch) sugar concentration was multiplied by the dry weight of the corresponding organs to get the sugar pool of the leaf (or branch); The leaf (or branch) starch and NSC pools were calculated using the same methods as the sugar pool. The root sugar (or starch and NSC) pool was estimated by multiplying the root dry weight by the root sugar (or starch and NSC) concentration. It was more challenging to calculate the stem NSC pool. In order to compute the NSC pool for each wood segment, the average NSC concentrations for H1 and H2, H2 and H3, H3 and H4, and H4 and H5 were determined separately. The average NSC concentration was then multiplied by the corresponding wood segment’s dry weight to get the NSC pool. The NSC pools of the complete stem could be obtained by summing the NSC pools of the five wood segments.

The dataset had a hierarchical structure with organs nested within trees that were themselves clustered within plots. Mutual dependence of the measurements had to be regarded in the statistical analysis. Thus, an appropriate statistical analysis of the dataset had to combine the concept of generalized models together with the multilevel and mixed model approach. Organ sugar (or starch and NSC) concentration was used as the response variable, fertilization regimes and height (or branch position and root diameter) were used as independent variables, and the plot was used as a covariate to construct a linear mixed model. In model building, independent fixed variables and their different combinations describing properties of the plots, trees and organs were tested for the spatial variations of organ sugar (or starch and NSC) concentration. Variables were included into the model at a 0.05 level of significance.

A univariate regression model was used to investigate the relationship between each organ’s biomass (independent variable) and NSC (including sugar and starch) pool (dependent variable). Residual analysis confirmed the dependent variable’s ability to fit. Multiple comparisons of tree DBH growth undergoing different fertilization regimes were performed using the Bonferroni method. The difference between fertilization was significant if the P value was less than 0.05.

## Results

### Variation of DBH growth

**Figure 1:**
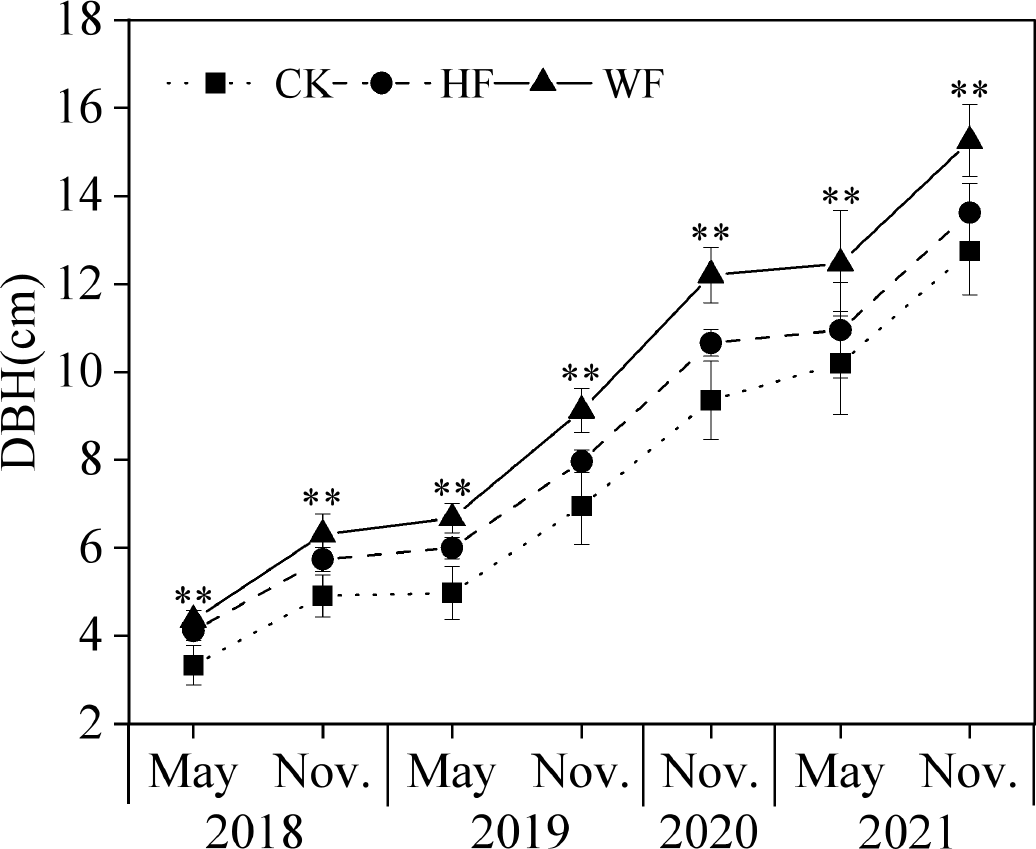
The DBH dynamics of *C. bungei* with different fertilization regimes from 2018 to 2021. CK, no fertilization (square pattern with dot); HF, hole fertilization (circle pattern with dash line); WF, integration of water and fertilizer (triangle pattern with solid line); DBH, diameter at breast height; **, *P*<0.01.

The DBH considerably varied between various fertilization schedules (**Figure 1**). The ranking of DBH from the largest to smallest was WF, HF, and CK. The DBH of HF and WF in November 2021 increased by 6.9% and 19.7% in comparison to CK, respectively. The DBH of the CK, HF, and WF increased by 282.9%, 231.2%, and 249.5% from 2018 to 2021, respectively.

### Longitudinal variation of leaf NSC concentration

**Figure 2:**
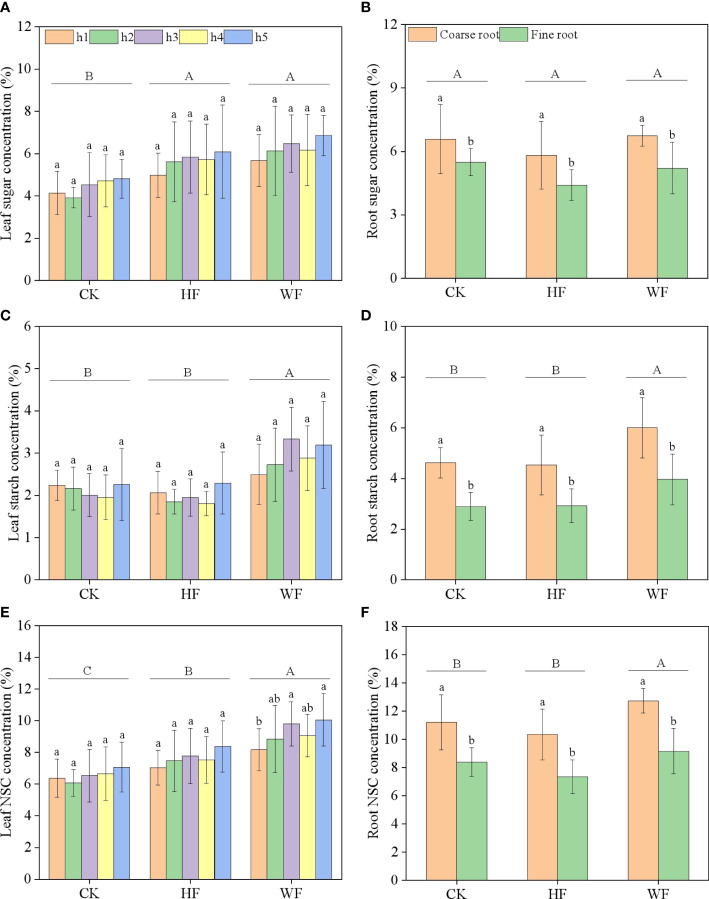
Longitudinal variation of leaf NSC concentration **(A–C)** and classification variation of root NSC concentration **(D–F)** with different fertilization regimes. CK, no fertilization. HF, hole fertilization. WF, integration of water and fertilizer. Different capital letters in the figure indicated significant differences between fertilization (*P*<0.05). Different lowercase letters indicated significant differences between height (or classification) (*P*<0.05).

The concentrations of sugar and starch in the leaves were unaffected by height or its interaction with fertilization (**Table 1**). The three fertilization techniques had similarly vertical distribution patterns for sugar and NSC concentrations. The concentrations of sugar and NSC tended to rise gradually with height (**Figures 2A, C**). Fertilization considerably altered the amounts of sugar, starch, and NSC in the leaves (**Table 1**). WF obviously had larger quantities of starch and NSC than HF and CK did (**Figures 2B, C**). When compared to CK, the sugar concentrations of HF and WF rose by 27.8% and 41.7%, respectively (**Table S3**). The NSC concentrations of HF and WF rose by 16.8% and 40.4% in comparison to CK, respectively.

**Table 1:** Analysis of variance of the NSC concentration in the leaf, branch, stem, bark, and root.

Variables	Sugar			Starch			NSC		
	df	*F*	*P*	df	*F*	*P*	df	*F*	*P*
**Leaf**									
Fertilization	2/10	8.346	**0.008**	2/14	9.765	**0.002**	2/10	16.939	**0.001**
Height	4/72	1.815	0.135	4/77	1.558	0.194	4/74	3.024	**0.023**
Fertilization×Height	8/72	0.160	0.995	8/77	1.091	0.379	8/74	0.346	0.945
**Branch**									
Fertilization	2/5	1.302	0.358	2/3	2.496	0.244	2/4	0.218	0.813
Height	4/343	0.725	0.575	4/343	1.056	0.117	4/343	2.123	0.078
Position	3/343	2.223	0.085	3/343	4.352	**0.005**	3/343	2.451	0.063
Fertilization×Height	8/343	0.362	0.940	8/343	0.905	0.512	8/343	0.510	0.849
Fertilization×Position	6/343	0.320	0.927	6/343	0.770	0.594	6/343	0.496	0.811
**Stem**									
Fertilization	2/90	8.295	**0.000**	2/134	2.503	0.086	2/18	7.516	**0.004**
Height	4/90	5.920	**0.000**	4/74	1.353	0.234	4/72	6.006	**0.000**
Fertilization×Height	8/90	0.791	0.612	8/74	1.618	0.134	8/72	1.360	0.229
**Bark**									
Fertilization	2/18	8.262	**0.003**	2/4	6.440	0.056	2/19	10.572	**0.001**
Height	4/76	43.723	**0.000**	4/72	35.271	**0.000**	4/72	56.457	**0.000**
Fertilization×Height	8/76	1.414	0.204	8/72	1.798	0.091	8/72	1.676	0.119
**Root**									
Fertilization	2/4	2.653	0.189	2/3	7.494	**0.044**	2/4	8.102	**0.036**
Classification	1/34	16.893	**0.000**	1/29	49.726	**0.000**	1/32	58.625	**0.000**
Fertilization×Classification	2/34	0.164	0.849	2/29	0.246	0.784	2/32	0.304	0.740

Fertilization referred to CK (no fertilization), HF (hole fertilization), WF (integration of water and fertilizer). Classification referred to coarse root and fine root. Position referred to different sampling sites of branch in the horizontal direction. The bold values in the table showed significant difference (P<0.05).

### Classification variation of root NSC concentration

Fertilization had no effect on sugar concentrations in the roots, but it did have an impact on starch and NSC levels (**Table 1**). When compared to CK and HF, the starch concentration in WF rose by 32.5% and 33.5%, respectively (**Table S3**). The NSC concentration of WF rose by 11.8% and 23.9% in comparison to the CK and HF, respectively. Varying diameters of roots have considerably different sugar, starch, and NSC concentrations (**Table 1**). In comparison to fine roots, coarse roots had significantly larger quantities of sugar, starch, and NSC (**Figures 2D–F**).

### Longitudinal and horizontal variation of branch NSC concentration

**Figure 3:**
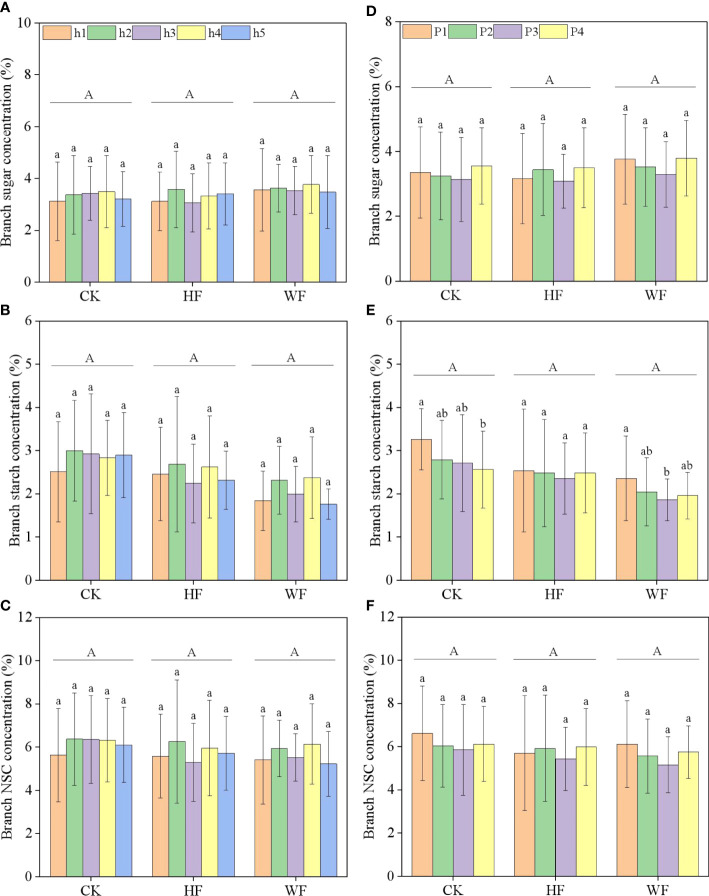
Longitudinal variation **(A–C)** and horizontal variation **(D–F)** of the branch NSC concentration with different fertilization regimes. CK, no fertilization. HF, hole fertilization. WF, integration of water and fertilizer. Different capital letters in the figure indicated significant differences between fertilization (*P*<0.05). Different lowercase letters indicated significant differences between height (or position) (*P*<0.05).

The concentrations of branch sugar, starch, and NSC were unaffected by height or its interaction with fertilization (**Table 1**; **Figures 3A–C**). The location or its interactions with fertilization had little impact on the concentrations of sugar and NSC (**Figures 3D–F**). The starch concentration steadily dropped from the branch’s base to its tip in three fertilization regimes (**Figure 3E**). Fertilization did not significantly influence sugar, starch, and NSC concentrations of the branch (**Table 1**).

### Longitudinal variation of stem NSC concentration

**Figure 4:**
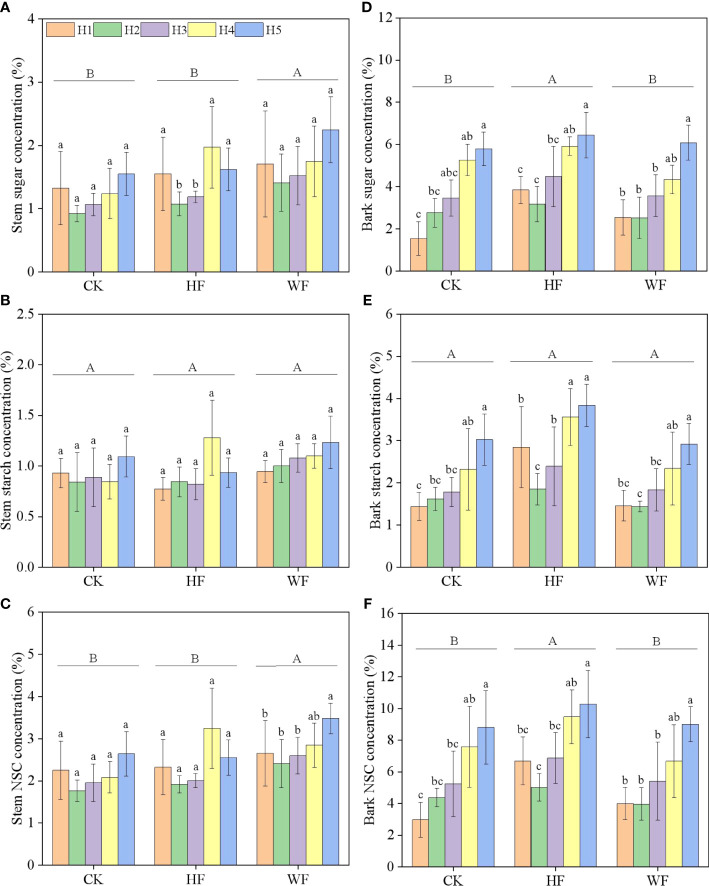
Longitudinal variation of the stem **(A–C)** and bark **(D–F)** NSC concentration with different fertilization regimes. CK, no fertilization. HF, hole fertilization. WF, integration of water and fertilizer. Different capital letters in the figure indicated significant differences between fertilization (*P*<0.05). Different lowercase letters indicated significant differences between height (*P*<0.05).

The concentrations of stem sugar and NSC were significantly impacted by fertilization and height, but not starch (**Table 1**). The concentrations of sugar and NSC first fell and then climbed with height in various fertilization regimens (**Figures 4A, C**). When compared to CK and HF, the sugar concentration in WF rose by 41.4% and 16.6%, respectively. (**Table S3**). The NSC concentration of WF increased by 30.7% and 16.1% in comparison to CK and HF, respectively.

### Longitudinal variation of bark NSC concentration

The sugar and NSC concentrations of the bark were significantly influenced by height and fertilization (**Table 1**). Height was correlated with higher amounts of sugar, starch, and NSC in CK and WF (**Figures 4D–F**). The concentrations of sugar, starch, and NSC undergoing HF initially decreased with height, then climbed. When compared to CK and WF, the sugar concentration in HF increased by 26.7% and 25.3%, respectively (**Table S3**). The NSC concentration of HF rose by 32.1% and 32.0% in comparison to CK and WF, respectively.

### Variation of organ NSC pool

**Figure 5:**
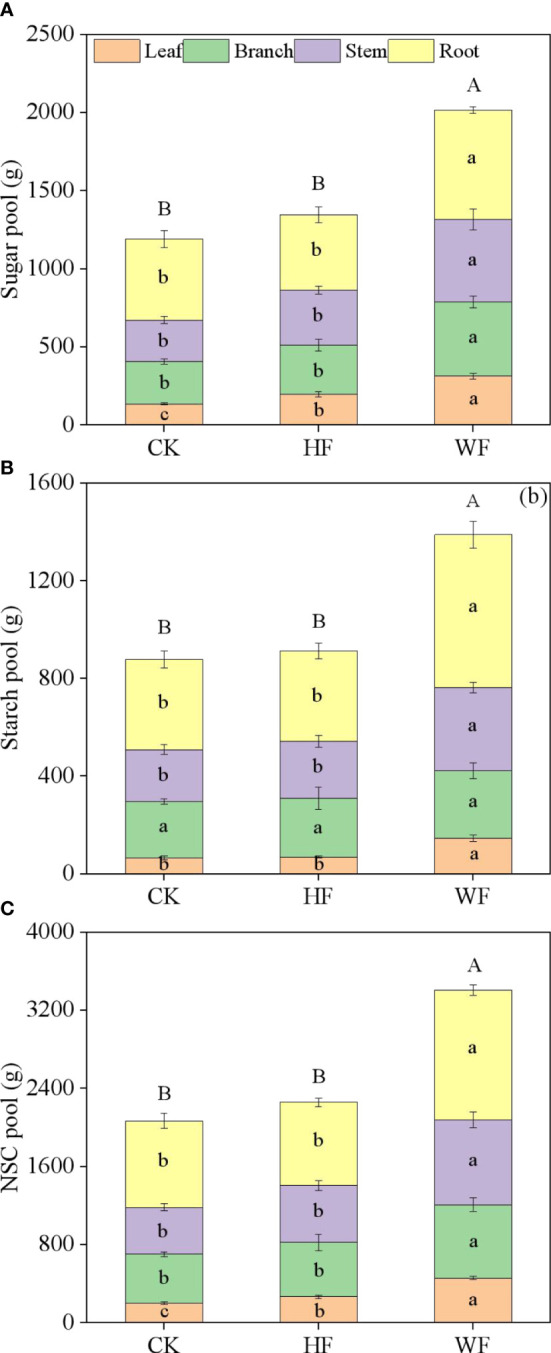
Variation of organ NSC pools with different fertilization regimes. CK, no fertilization. HF, hole fertilization. WF, integration of water and fertilizer, **(A)** sugar pool, **(B)** starch pool, **(C)** NSC pool. Different lowercase letters in the same organ indicated significant differences between fertilization (*P*<0.05). Different capital letters in the whole tree indicated significant differences between fertilization (*P*<0.05).

WF had much larger sugar, starch, and NSC pools than CK and HF did in each organ (**Figures 5A–C**). The whole-tree undergoing WF had considerably greater sugar, starch, and NSC pools than CK and HF. In comparison to CK and HF on the entire tree, the WF increased by 69.4% and 50.04% (for sugar), 58.34% and 52.14% (for starch), and 64.7% and 50.84% (for NSC), respectively (**Table S4**). The above-ground organs’ sugar, starch, and NSC pools were higher than those of the below-ground organs in various fertilization regimes.

The distribution ratios of sugar, starch, and NSC pools in the same organ varied when three distinct fertilization strategies were used. The HF and WF enhanced the sugar pool distribution ratio in the leaves and stems while decreasing it in the roots in comparison to CK (**Table S5**). When compared to CK and HF, the WF increased the fraction of starch pools in the leaves and roots while decreasing it in the branches. The HF and WF increased the amount of NSC pools in the leaves and stems while decreasing the proportion in the roots in comparison to CK.

### Simulation of the relationship between NSC pools and organ biomass

The relationship between each organ’s biomass and its related sugar, starch, and NSC pools was fit using a univariate regression equation. R^2^ for all models ranged from 0.343 to 0.833 (**Table 2**). As leaf biomass rose, the leaf sugar, starch, and NSC pools steadily grew as well (**Figures S3A–C**). Branch, stem, and root alteration patterns resembled those of leaves (**Figures S3D–F**; **Figure S4**). The fitting effects of all models were perfect since the residual distribution was comparatively homogeneous (**Figures S5, S6**).

**Table 2:** Univariate linear equation established by sugar, starch, and NSC pools and biomass in the leaf, branch, stem, and root.

Organ	Equation	RSS	r	Adjust R^2^	F	*P*
Leaf						
Sugar pool−biomass	y=-48.044+23.194x	39171.859	0.818	0.649	32.400	<0.001
Starch pool−biomass	y=-52.098+12.871x	8017.671	0.868	0.737	48.748	<0.001
NSC pool−biomass	y=-100.143+36.065x	47397.092	0.895	0.789	64.743	<0.001
Branch						
Sugar pool−biomass	y=-19.840+18.728x	52156.461	0.881	0.762	55.385	<0.001
Starch pool−biomass	y=73.909+8.789x	64430.426	0.618	0.343	9.875	0.006
NSC pool−biomass	y=54.068+27.517x	180910.131	0.826	0.663	34.472	<0.001
Stem						
Sugar pool−biomass	y=-323.960+14.539x	82020.201	0.886	0.771	58.298	<0.001
Starch pool−biomass	y=-99.243+7.431x	20879.641	0.888	0.776	59.815	<0.001
NSC pool−biomass	y=-423.202+21.970x	127103.328	0.918	0.833	85.899	<0.001
Root						
Sugar pool−biomass	y=-3.231+22.760x	175948.948	0.701	0.460	15.456	0.001
Starch pool−biomass	y=-214.378+26.749x	181951.363	0.751	0.536	20.644	<0.001
NSC pool−biomass	y=-217.610+49.509x	360809.032	0.831	0.671	35.663	<0.001

y referred to sugar, starch, and NSC pools. x referred to biomass of the leaf, branch, stem, and root. RSS referred to residual sum of squares. r referred to correlation coefficient.

## Discussion

### Longitudinal variation of leaf NSC concentration

The results of *Pinus koraiensis* (Yan et al., 2012), *Pinus cerebra* (Li et al., 2001), *Abies georgei* and *Sabina saltuaria* (Li et al., 2009) were partially consistent with the findings of this study in that the concentrations of sugar and starch in the leaf were not significantly influenced by canopy height. Although we thought the upper crown had a large capability for photosynthetic activity, the strong output capacity of the upper crown meant that there was little NSC variation among the crown layers. Our findings that fertilization may increase the leaf’s NSC concentration were corroborated by the observations of Wei et al. (2021), which revealed that nitrogen and phosphorus input could raise the leaf’s net photosynthetic rate, which in turn increased the generation of organic carbon and raised the leaf’s NSC concentration.

### Spatial variation of branch NSC concentration

Our investigation found that the sugar, starch, and NSC concentrations of the branch did not change considerably at five crown heights, which was consistent with the findings of *Fagus sylvatica* and *Quercus petraea* (Barbaroux et al., 2003). According to Woodruff and Meinzer (2011), the moisture did not significantly affect the transit of phloem NSC from the upper crown to the trunk and root, and the higher crown’s stronger carbon source may have compensated for the effect of carbon sinks. The NSC concentrations of *Pinus ponderosa* and *Pseudotsuga menziesii* branch, however, rose with height (Sala and Hoch, 2008; Woodruff and Meinzer, 2011). Coniferous and broad-leaved trees in particular, which had different living habits, anatomical traits, and survival strategies, may be to blame for the gap. Our findings demonstrated that despite a small difference in the branch’s NSC content along its horizontal axis, the starch concentration declined steadily from the branch’s base to its tip. These results were inconsistent with the reports of Zhang et al. (2015), who discovered that the increased tissue vitality from the center to outer part of the crown was responsible for the branch NSC concentration steadily increasing from the base to tip of the branch. The fact that the branch in our experiment had its bark removed could be the cause of the discrepancy. Actually, the branch’s bark or lack thereof might have an impact on the NSC pools and the distribution ratio of sugar and starch (Barbaroux et al., 2003).

### Longitudinal variation of stem and bark NSC concentration

Our findings revealed that the sugar and NSC concentrations of stem initially fell and then progressively increased with height, which was somewhat consistent with the reports of *Quercus petraea* and *Fagus sylvatica* (Barbaroux et al., 2003), *Betula platyphylla* and *Tilia amurensis* (Zhang et al., 2014). Sala et al. (2012) discovered that while the upper stem was more susceptible to embolism, it tended to store more NSC (particularly soluble carbohydrates) to prevent embolism (Ameglio et al., 2001; Woodruff and Meinzer, 2011; Nardini et al., 2011; Sala et al., 2012). We noticed that the NSC concentration of *C. bungei* stem (2.140%-2.798%; **Table S3**) were within the range of previous reports of the temperate forests (0.2%-9.0%; Barbaroux and Bréda, 2002; Barbaroux et al., 2003; Hoch et al., 2003; Gough et al., 2009), but primarily lower than those of the tropical forests (3.0-30.0%; most species about 10.0%; Newell et al., 2002; Würth et al., 2005), indicating that the leaf phenology and wood types of tree species affected the distribution strategy of stem NSC. We discovered that the concentrations of sugar, starch, and NSC in the bark steadily increased with height undergoing water and fertilizer integration, indicating that the levels of bark NSC appeared to be more plentiful in the upper regions of the crown. Additionally, the NSC concentration in the bark was significantly higher than that in the stem (**Table S3**), which was in line with the findings of Barbaroux and Bréda (2002).

### Variation of root NSC concentration

Barbaroux et al. (2003) discovered that the NSC concentrations rose with root diameter in *Quercus petraea* and *Fagus sylvatica* roots. In addition, Yu et al. (2011) noted that the fine roots of *Quercus mongolica* had a lower NSC concentration than the medium roots and coarse roots. Our research only partially supported these findings. Despite the fact that fertilization had no discernible effect on the sugar concentration in the roots compared to no fertilization, the combination of water and fertilizer increased the starch concentration of the roots, particularly coarse roots, indicating that the substantial amounts of starch stored in the roots could be used to maintain respiration during the dormancy and springtime growth (Wargo, 1976).

### Variation of organ NSC pool

Our findings supported the reports of Loeschner et al. (1990), who hypothesized that the roots were more highly specialized as storage organs than any other organ and that the NSC pool primarily dominated the roots. In our trees, the contributions of the various organs - roots (40.0%), stems (25.0%), branches (23.7%), and leaves (11.7%) - to total NSC storage varied. At the end of the growing season, the *C. bungei* NSC pool’s distribution strategy would assist the tree withstand the cold. The organ-level storage, however, varies between species. According to Barbaroux et al. (2003), the main NSC reservoir with the greatest amount of biomass was thought to be the stem of *Quercus petraea* and *Fagus sylvatica*. The leaves of *Picea abies* and *Pinus cembra* (Oren et al., 1988; Gruber et al., 2011), as well as the roots of *Pinus elliottii* (Gholz and Cropper, 1991) and *Pinus koraiensis* (Yu et al., 2011), were said to have exceptionally big sugar pools, starch pools, and NSC pools, respectively, according to certain researchers. Thus, the growth traits, survival strategy, and cold tolerance of tree species might account for the disparities in NSC pool dispersion. The total tree’s NSC pool in this investigation ranged from 2.1 kg to 3.4 kg (**Table S4**), which is significantly less than the values obtained from *Quercus rubra*, *Pinus strobus*, *Acer rubrum*, *Betula papyrifera*, and *Fraxinus americana* (10-50 kg; Furze et al., 2019). The discrepancy was caused by the different age, size, leaf habit, and wood anatomy of the investigated tree species. For instance, previous research frequently claimed that deciduous species had higher storage requirements than evergreen species (Kozlowski, 1992; Hoch et al., 2003). Similar disparities in storage were also seen based on the anatomy of the wood, with ring-porous species having bigger reserves than diffuse-porous species (Barbaroux and Breda, 2002). A more reliable estimate of total NSC storage at the ecosystem level was facilitated by these findings, which also served as the impetus for measurement of whole-tree total NSC storage across several species.

Our data showed that the DBH growth with integrated water and fertilizer application was significantly higher than undergoing hole application and no fertilization from 2018 to 2021 (**Figure 1**). In addition, the NSC pool of the whole-tree undergoing integrated water and fertilization and hole fertilization grew by 64.7% and 9.2% in comparison to no fertilization, respectively (**Table S4**). The integration of water and fertilizer might increase their efficiency of use, increase the root system’s absorption vitality, increase the capacity of leaves for photosynthetic absorption, and ultimately stimulate plant development and the accumulating of carbon pools (Zou et al., 2020; Fan et al., 2020; Sun et al., 2022). Thus, prioritizing the integration of water and fertilizer will boost the biomass and carbon storage capacity of trees. A univariate regression equation was utilized to determine the relationship between NSC pools and organ biomass. This equation could be used to forecast the carbon pools of the leaves, branches, stems, and roots, hence calculating the amount of carbon stored by the entire tree or stand. However, the constructed models had certain drawbacks because of tiny sample size (18 trees) in our experiment. Future study should examine various age stages of tree growth from the standpoint of extending the regional scale and increasing the sample size to create a more precise prediction model of carbon storage in the *C. bungei*.

## Conclusions

Our findings painted a picture of NSC storage in the *C. bungei* that included the following: (1) Different fertilization regimes resulted in different sizes of whole-tree total NSC pools. (2) The fertilization plans had little to no impact on the spatial distribution patterns of the concentrations of organ sugar, starch, and NSC. (3) The roots were the primary organ of storage for NSC pools. In contrast to no fertilization and hole fertilization, the combination of water and fertilizer considerably boosted tree growth and carbon storage, which was deserving of attention in further fertilization studies. Our findings therefore enhanced our comprehension of C dynamics at the whole-tree level and, more crucially, clarified how the dynamics of different organs affected the overall C balance.

## Data availability statement

The original contributions presented in the study are included in the article/**Supplementary Material**. Further inquiries can be directed to the corresponding authors.

## Author contributions

ZG and QL conceived and designed the experiments. YuL, DL, YiL, QinH, and NL conducted laboratory analyses. WM, YS, JL, and JW analyzed the data and wrote results. QQ and QinH wrote the manuscript (Introduction and Discussion); all authors provided editorial advice and revised manuscript. All authors read and approved the final manuscript

## Funding

This research was supported by the grants from National Key Research and Development Program of China (2017YFD0600604, 2017YFD060060404).

## Acknowledgments

We gratefully acknowledge Professor Qian He and Quan Qiu for their advice of field experiment design and this manuscript.

## Conflict of interest

The authors declare that the research was conducted in the absence of any commercial or financial relationships that could be construed as a potential conflict of interest.

## Publisher’s note

All claims expressed in this article are solely those of the authors and do not necessarily represent those of their affiliated organizations, or those of the publisher, the editors and the reviewers. Any product that may be evaluated in this article, or claim that may be made by its manufacturer, is not guaranteed or endorsed by the publisher.
