# Supplementary material for: Spatial variations and pools of non-structural carbohydrates in young Catalpa bungei undergoing different fertilization regimes
Source: Front Plant Sci. 2022 Sep 29;13:1010178. doi: 10.3389/fpls.2022.1010178 (PMC9557222; doi:10.3389/fpls.2022.1010178)
Supplement: Supplementary file 1 [file DataSheet_1.docx]

**Fig. S1** Monthly precipitation (dark bars) and mean temperature (circle pattern with dotted line) from 2017 to 2021 in Zhangqiu District, Jinan City, China.


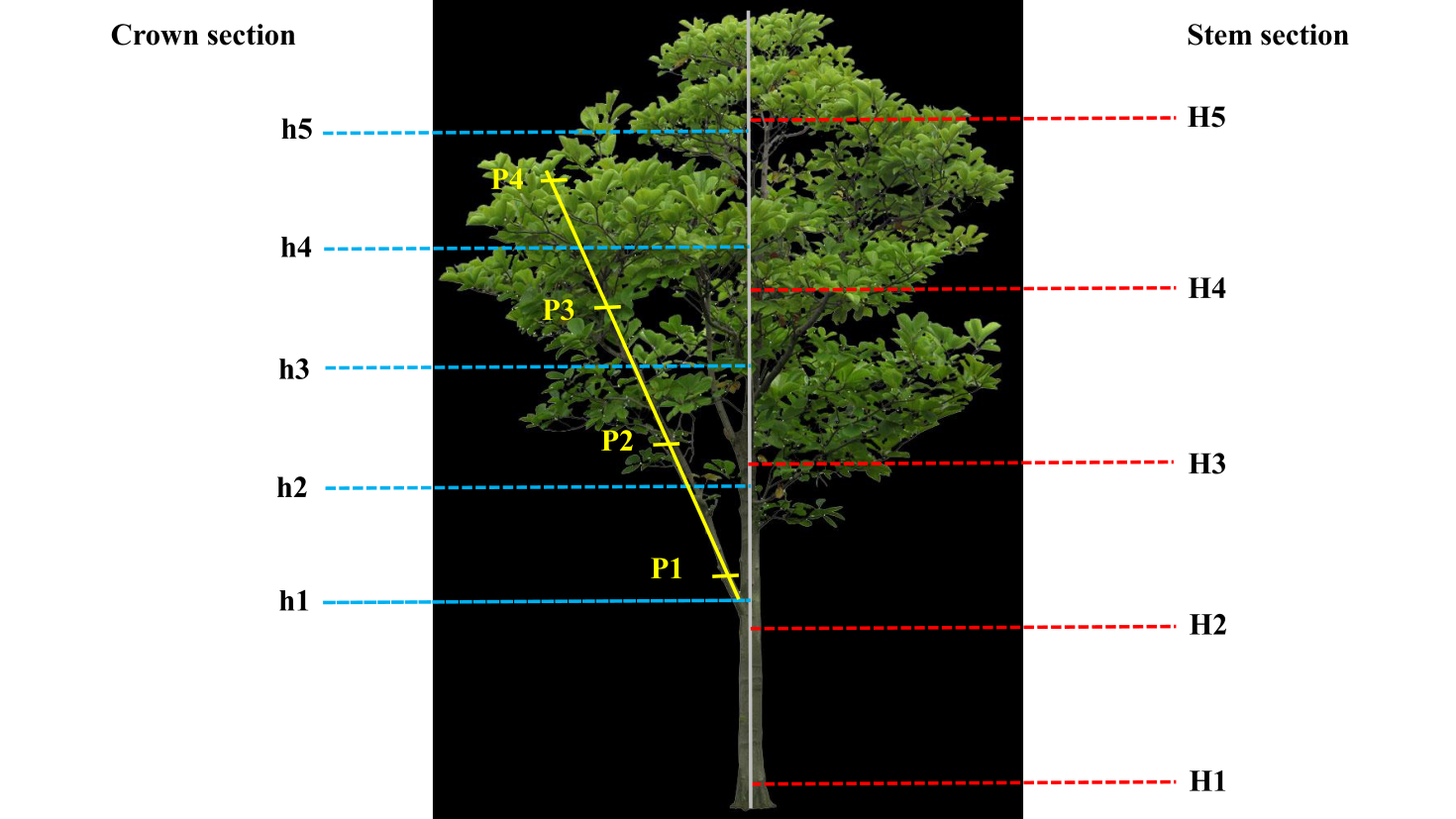


**Fig. S2** Schematic diagram of five equal parts of *Catalpa bungei* stem and crown.

**Fig.S3** Relationship between the leaf (a-c) and branch (d-f) NSC pools and biomass.

**Fig.S4** Relationship between the stem (a-c) and root (d-f) NSC pools and biomass.

**Fig. S5** Residual distribution of the leaf (a-c) and branch (d-f) NSC pools.

**Fig.S6** Residual distribution of stem (a-c) and root (d-f) NSC pools.
