# Supplementary material for: Spatial variations and pools of non-structural carbohydrates in young Catalpa bungei undergoing different fertilization regimes
Source: Front Plant Sci. 2022 Sep 29;13:1010178. doi: 10.3389/fpls.2022.1010178 (PMC9557222; doi:10.3389/fpls.2022.1010178)
Supplement: Supplementary file 2 [file DataSheet_2.docx]

**Table S1** Growth index with different fertilization in October 2021 for *[C.](D:/Dict/8.9.6.0/resultui/html/index.html#/javascript:;) [bungei](D:/Dict/8.9.6.0/resultui/html/index.html#/javascript:;)* plantation.

| Fertilization | Tree height (m) | |  | DBH (cm) | |  | Crown diameter (m) | |
| --- | --- | --- | --- | --- | --- | --- | --- | --- |
|  | Min.-Max. | Mean±SD |  | Min.-Max. | Mean±SD |  | Min.-Max. | Mean±SD |
| CK | 6.5-9.6 | 8.5±0.63 |  | 9.85-14.12 | 12.74±0.99 |  | 2.35-4.45 | 2.91±0.40 |
| HF | 8.1-9.7 | 8.9±0.41 |  | 12.41-14.89 | 13.62±0.66 |  | 2.65-4.40 | 3.22±0.37 |
| WF | 7.1-10.0 | 9.1±0.58 |  | 13.43-16.80 | 15.25±0.82 |  | 2.80-4.55 | 3.50±0.34 |

Note: CK, no fertilization; HF, hole fertilization; WF, [integration](D:/Dict/8.9.6.0/resultui/html/index.html#/javascript:;) [of](D:/Dict/8.9.6.0/resultui/html/index.html#/javascript:;) [water](D:/Dict/8.9.6.0/resultui/html/index.html#/javascript:;) [and](D:/Dict/8.9.6.0/resultui/html/index.html#/javascript:;) [fertilizer](D:/Dict/8.9.6.0/resultui/html/index.html#/javascript:;); DBH, diameter at breast height; SD, [standard](D:/Dict/8.9.6.0/resultui/html/index.html#/javascript:;) [deviation](D:/Dict/8.9.6.0/resultui/html/index.html#/javascript:;).

**Table S2** Growth index with different fertilization for felled sample trees

| Fertilization | Tree height (m) | |  | DBH (cm) | |  | Crown diameter (m) | |
| --- | --- | --- | --- | --- | --- | --- | --- | --- |
|  | Min.-Max. | Mean±SD |  | Min.-Max. | Mean±SD |  | Min.-Max. | Mean±SD |
| CK | 8.2-9.3 | 9.0±0.42 |  | 11.78-13.20 | 12.57±0.62 |  | 2.35-3.91 | 3.15±0.64 |
| HF | 8.7-9.5 | 9.2±0.30 |  | 13.08-13.95 | 13.55±0.35 |  | 2.69-3.60 | 3.32±0.34 |
| WF | 9.0-10.4 | 9.7±0.53 |  | 14.31-15.72 | 15.02±0.56 |  | 3.26-4.14 | 3.69±0.31 |

Note: CK, no fertilization; HF, hole fertilization; WF, [integration](D:/Dict/8.9.6.0/resultui/html/index.html#/javascript:;) [of](D:/Dict/8.9.6.0/resultui/html/index.html#/javascript:;) [water](D:/Dict/8.9.6.0/resultui/html/index.html#/javascript:;) [and](D:/Dict/8.9.6.0/resultui/html/index.html#/javascript:;) [fertilizer](D:/Dict/8.9.6.0/resultui/html/index.html#/javascript:;); DBH, diameter at breast height; SD, [standard](D:/Dict/8.9.6.0/resultui/html/index.html#/javascript:;) [deviation](D:/Dict/8.9.6.0/resultui/html/index.html#/javascript:;).

**Table S3** Average NSC concentration of each organ with different fertilization regimes.

| Organ | CK |  | HF |  | WF |
| --- | --- | --- | --- | --- | --- |
|  | Mean±SD |  | Mean±SD |  | Mean±SD |
| **Sugar concentration (%)** |  |  |  |  |  |
| Leaf | 4.419±1.070 B |  | 5.646±1.664 A |  | 6.260±1.463 A |
| Branch | 3.322±1.305 A |  | 3.299±1.238 A |  | 3.591±1.203 A |
| Stem | 1.221±0.401 B |  | 1.481±0.646 B |  | 1.727±0.617 A |
| Bark | 3.766±2.107 B |  | 4.771±1.537 A |  | 3.809±1.840 B |
| Root | 6.040±1.308 A |  | 5.110±1.398 A |  | 5.975±1.196 A |
| **Starch concentration (%)** |  |  |  |  |  |
| Leaf | 2.123±0.549 B |  | 1.991±0.480 B |  | 2.926±0.985 A |
| Branch | 2.832±1.119 A |  | 2.464±1.116 A |  | 2.055±0.741 A |
| Stem | 0.920±0.232 A |  | 0.930±0.422 A |  | 1.072±0.185 A |
| Bark | 2.037±0.787 A |  | 2.898±1.247 A |  | 2.000±0.753 A |
| Root | 3.758±1.057 B |  | 3.730±1.245 B |  | 4.978±1.491 A |
| **NSC concentration (%)** |  |  |  |  |  |
| Leaf | 6.542±1.369 C |  | 7.638±1.551 B |  | 9.186±1.634 A |
| Branch | 6.154±1.991 A |  | 5.763±2.132 A |  | 5.647±1.608 A |
| Stem | 2.140±0.540 B |  | 2.411±1.003 B |  | 2.798±0.634 A |
| Bark | 5.803±2.783 B |  | 7.668±2.474 A |  | 5.810±2.494 B |
| Root | 9.798±2.096 B |  | 8.840±2.148 B |  | 10.953±2.238 A |

Note: CK, no fertilization; HF, hole fertilization; WF, [integration](D:/Dict/8.9.6.0/resultui/html/index.html#/javascript:;) [of](D:/Dict/8.9.6.0/resultui/html/index.html#/javascript:;) [water](D:/Dict/8.9.6.0/resultui/html/index.html#/javascript:;) [and](D:/Dict/8.9.6.0/resultui/html/index.html#/javascript:;) [fertilizer](D:/Dict/8.9.6.0/resultui/html/index.html#/javascript:;); Different capital letters in the same row indicated significant difference between fertilization (*P*<0.05).

**Table S4** NSC pools of the above-ground organs, below-ground organs, and whole trees.

| Fertilization | CK | HF | WF |
| --- | --- | --- | --- |
| **Above-ground organs (g)** |  |  |  |
| Sugar | 670.81±79.01c | 862.07±128.52b | 1314.46±285.29a |
| Starch | 508.02±77.84b | 541.12±152.41b | 762.13±103.84a |
| NSC | 1178.83±150.06b | 1403.18±270.85b | 2076.58±372.58a |
| **Below-ground organs (g)** |  |  |  |
| Sugar | 518.28±135.80b | 481.40±123.18b | 700.30±51.04a |
| Starch | 368.86±82.76b | 371.38±78.59b | 625.92±134.03a |
| NSC | 887.14±190.23b | 852.78±107.94b | 1326.22±133.86a |
| **Whole-tree (g)** |  |  |  |
| Sugar | 1189.09±185.83b | 1343.46±210.88b | 2014.75±325.43a |
| Starch | 876.88±126.60b | 912.51±147.11b | 1388.05±199.79a |
| NSC | 2065.97±283.62b | 2255.96±315.47b | 3402.80±482.88a |

Note: CK, no fertilization; HF, hole fertilization; WF, [integration](D:/Dict/8.9.6.0/resultui/html/index.html#/javascript:;) [of](D:/Dict/8.9.6.0/resultui/html/index.html#/javascript:;) [water](D:/Dict/8.9.6.0/resultui/html/index.html#/javascript:;) [and](D:/Dict/8.9.6.0/resultui/html/index.html#/javascript:;) [fertilizer](D:/Dict/8.9.6.0/resultui/html/index.html#/javascript:;); Different lowercase letters in the same row indicated significant differences between fertilization (*P*<0.05). The above-ground organs referred to the sum of leaves, branches, and stems.The below-ground organs referred to the sum of coarse roots and fine roots.

**Table S5** Percentage of sugar, starch, and NSC pools allocation to each organ undergoing different fertilization.

|  | CK | |  | HF | |  | WF | |
| --- | --- | --- | --- | --- | --- | --- | --- | --- |
| Sugar pool (g) | Mean±SD | Ratio (%) |  | Mean±SD | Ratio (%) |  | Mean±SD | Ratio (%) |
| Leaf | 133.01±6.83 | 11.19 |  | 195.24±16.42 | 14.53 |  | 310.95±19.28 | 15.43 |
| Branch | 270.77±16.40 | 22.77 |  | 315.24±37.96 | 23.46 |  | 474.55±38.14 | 23.55 |
| Stem | 267.03±24.95 | 22.46 |  | 351.58±26.31 | 26.17 |  | 528.95±65.07 | 26.25 |
| Root | 518.28±55.44 | 43.59 |  | 481.40±50.29 | 35.83 |  | 700.30±20.84 | 34.76 |
| Whole-tree | 1189.09±75.86 | 100.00 |  | 1343.46±86.09 | 100.00 |  | 2014.75±132.86 | 100.00 |
|  |  |  |  |  |  |  |  |  |
| Starch pool (g) | Mean±SD | Ratio (%) |  | Mean±SD | Ratio (%) |  | Mean±SD | Ratio (%) |
| Leaf | 64.56±6.89 | 7.36 |  | 68.66±3.00 | 7.52 |  | 145.19±14.14 | 10.46 |
| Branch | 231.08±10.24 | 26.35 |  | 240.00±45.50 | 26.30 |  | 276.33±32.68 | 19.91 |
| Stem | 212.38±20.23 | 24.22 |  | 232.47±23.93 | 25.48 |  | 340.61±20.33 | 24.54 |
| Root | 368.86±33.79 | 42.07 |  | 371.38±32.09 | 40.70 |  | 625.92±54.72 | 45.09 |
| Whole-tree | 876.88±51.69 | 100.00 |  | 912.51±60.06 | 100.00 |  | 1388.05±81.57 | 100.00 |
|  |  |  |  |  |  |  |  |  |
| NSC pool (g) | Mean±SD | Ratio (%) |  | Mean±SD | Ratio (%) |  | Mean±SD | Ratio (%) |
| Leaf | 197.57±13.27 | 9.56 |  | 263.89±17.17 | 11.70 |  | 456.15±16.94 | 13.41 |
| Branch | 501.85±23.48 | 24.29 |  | 555.24±83.12 | 24.61 |  | 750.87±68.46 | 22.07 |
| Stem | 479.42±37.50 | 23.21 |  | 584.05±48.69 | 25.89 |  | 869.56±83.03 | 25.55 |
| Root | 887.14±77.66 | 42.94 |  | 852.78±44.07 | 37.80 |  | 1326.22±54.65 | 38.97 |
| Whole-tree | 2065.97±115.79 | 100.00 |  | 2255.96±128.79 | 100.00 |  | 3402.80±197.14 | 100.00 |
